# Socio-epidemiological study of bovine brucellosis: Seasonal cattle migration in Myanmar

**DOI:** 10.5455/javar.2025.l903

**Published:** 2025-04-16

**Authors:** Su Su Hlaing, Hiroichi Kono

**Affiliations:** 1 Graduate School of Animal and Veterinary Sciences and Agriculture, Obihiro University of Agriculture and Veterinary Medicine, Obihiro, Japan.; 2 Department of Agro-Environmental Science, Obihiro University of Agriculture and Veterinary Medicine, Obihiro, Japan.

**Keywords:** Cattle migration, farmers’ characteristics, Bovine brucellosis, Myanmar

## Abstract

**Objective:**

Animal migration can spread different diseases from farm to farm without being noticed. Despite bovine brucellosis being endemic in Myanmar, seroprevalence and risk related to farmers’ behaviors and cattle migration remain unclear. Thus, understanding brucellosis in seasonal cattle migration is essential to avoid negative economic and public health outcomes. As seasonal cattle movement is done to satisfy local environmental limits, Brucellosis is predisposed in cattle herds of the Central Dry Zone of the country.

**Materials and Methods:**

In this study, farmer characteristics and seasonal cattle movement practices were investigated for bovine brucellosis prevalence in three Central Myanmar regions from July to August 2022. Blood samples were taken for the Rose Bengal Plate Test to detect brucellosis prevalence.

**Results:**

Seasonal migratory farmers had a lower education level, more family members, more cattle heads, and higher income from cattle raising. Influences on disease knowledge included frequent veterinary access (7.02%) and limited disease control training (17.39%). Young farmers with low educational level (*p* < 0.01), more family members (*p* < 0.01), less farming experience (*p* < 0.01), fewer cattle (*p* < 0.01), cow abortion cases (*p* < 0.01), farming practices from veterinary access (*p* < 0.01), and longer migratory distances to grazing pastures (*p* < 0.01) have higher possibilities to get prevalence of bovine brucellosis at farm level.

**Conclusion:**

This study found that farmers’ characteristics, migratory practices, migration distance, and abortion history influence Brucellosis prevalence at the farm level. Migratory farmers need farmer collaboration and veterinary training to learn effective farming practices. Access to veterinary services and farmer awareness campaigns about livestock migration risks are essential.

## Introduction

Myanmar’s economy and rural communities rely heavily on livestock, which provides both urban and rural populations with meat, milk, eggs, and financial assets. Water buffaloes, pigs, cattle, and poultry are among the main animals raised in Myanmar. Cattle and buffaloes continue to be a significant source of draft power for crop production in Myanmar, although many aspects of the country’s agricultural sector are mechanizing rapidly [1]. 30% of cattle and buffalo owners sell their animals, 15% utilize manure as fertilizer, and the majority raise their animals primarily for draft power [2].

In the Central Dry Zone (CDZ), 42% of households keep cattle for both seasonal migration and traditional backyard farming systems [1]. It is customary to relocate herds to fresh pastures and water according to the seasons. Perhaps, as a consequence of climate change, regular droughts in the CDZ increase the scarcity of forage resources, making competition severe for limited amounts of water and pasture. Therefore, when pastures and water are scarce, mobility remains the most important pastoral risk management tactic. Livestock losses are much lower for farmers who relocate herds after natural disasters [3]. However, because of competition for limited pasture and water, this mobility alone leads to conflicts among farmers.

Various infectious diseases are transmitted via livestock movement within the country and across borders, including anthrax, foot and mouth disease, rabies, brucellosis, tuberculosis, salmonellosis, and avian influenza. Smallholder farmers in developing countries face a significant risk of transboundary animal diseases (TADs) that threaten their economy, trade, and food security [4]. Unknown to farmers, animal movement is also known to contribute to the spread of disease [5]. Therefore, an adequate understanding of animal movement patterns is necessary to establish effective disease management strategies.

Due to its adverse impact on cow fertility and resulting zoonotic transmission, bovine brucellosis, endemic to Myanmar, continues to have a major economic and health impact. Premature abortions and breeding problems, including repeated breeding, retained products of conception, metritis, stillbirths, frailty in progeny, decreased production of milk in females, orchitis, and epididymitis in males, are characteristics of brucella infection [6]. By direct interaction with infected animals or indirect contact with contaminated animal products, human become unintentional hosts [7].

Using a more realistic animal movement pattern is crucial as infectious disease modeling has expanded in popularity as a method for estimating disease spread and evaluating the efficacy of control measures. Animal movement patterns have been examined and documented in certain countries, including Australia and the United Kingdom [8]. However, a number of biological, economic, environmental, and geographic factors can affect migratory patterns. To mitigate impacts on the economy and public health, new strategies for brucellosis control that take transboundary cattle migration into account are crucial. As such, understanding the influence of cattle migration in the spread of brucellosis results is crucial [9].

Limited knowledge about risk factors and seroprevalence results in the need to move the cattle seasonally to satisfy local environmental limits, making herds in the CDZ particularly prone to brucellosis. It is necessary to study the disease in different parts of Myanmar as previous research was limited to the country’s lower region [10]. The incidence of brucellosis in migratory cattle must be thoroughly understood to reduce its adverse impacts on the economy and public health. Currently, cattle migratory practices, access to veterinary services, and farmers’ perceptions of infectious cattle diseases remain poorly understood. In this study, given the high number of migratory farmers with large herds in the pastures of Myanmar’s CDZ, the farmers’ characteristics and seasonal cattle movement practices were investigated concerning the prevalence of bovine brucellosis in the area.

## Materials and Methods

### Ethical approval and informed consent

The study received approval from the Institutional Animal Ethics Committee, IAEC-UVS, University of Veterinary Science, Myanmar, dated April 20, 2022, by approval number 20220420115. Permission to publish the found data was given freely by the participating farm owners.

### Description of study area

**Figure 1.**
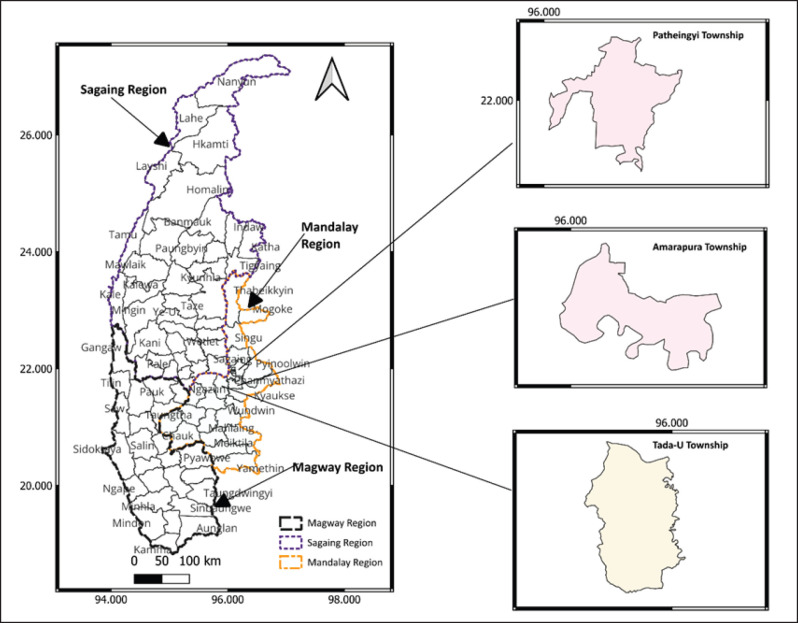
Map of the Central Dry Zone in Myanmar indicating the study areas: Patheingyi, Amarapura, and Tada-U Townships in Mandalay Region. Source: Author (2023).

This study was carried out in Myanmar’s CDZ, the Mandalay area (Fig. 1). The Dry Zone of Myanmar is located between latitudes 19°20” and 22°50” and longitudes 93°40” and 96°30” in the country’s center, east side of the Ayeyarwady River. Comprising 13 districts and 57 townships in the Sagaing, Mandalay, and Magway divisions, the dry zone occupies 33,680 square miles. The CDZ is inhabited by an estimated 12 million people, or 23% of Myanmar’s total population [11–13]. There are an estimated 9.7 million cattle and 1.8 million goats/sheep in the research region [14]. The two native breeds of cattle, Pyer Sein and Shwe Ni, are zebu type (*Bos indicus*), with a few Holstein–Friesian crossbreeds being the most prevalent. The herds consist primarily of animals with little artificial insemination, adapted to tropical climates, resistant to tropical diseases and external parasites, and able to survive on low-quality grasses and roughage [15]. Both semi-intensive and extensive management techniques have been taken into consideration for the study area. Many farmers practice agro-pastoralist farming, which involves raising small herds in their backyards, where the animals are fed grass from neighboring communal grazing spots. To procure sufficient food and water, farmers are accustomed to relocating the animals over long distances.

### Study design and sampling procedure

In 2022, a cross-sectional survey was carried out from June to August in the Amarapura, Patheingyi, and Tada-U townships of the CDZ (Fig. 1) to determine farmer characteristics related to seasonal cattle migration practices and their association with bovine brucellosis. With the help of the local veterinary department, a list of migratory cattle farmers in purposively selected areas and the time at which they were in the study sites was obtained. In total, 115 migratory farmers were selected based on their presence at the study sites. The study’s criteria for eligibility were willingness to participate in an interview and being head of household, spouse, or adult (at least 18 years old) family member, in the absence of the household head or spouse.

### Questionnaire survey

Structured questionnaires administered by interviewers were used to collect data on the farmers’ characteristics, migration practices, and brucellosis risk factors. For purposes of uniformity, clarity, and sociocultural consent in the community, the questionnaire was translated from the original English version into the local language (Myanmar) and then back into English. Additional data was acquired, and several of the questions were modified during pre-testing. Trained research assistants and the main investigator conducted interviews with the participants in the Myanmar language. The interviewers provided the household members with relevant disease information once they had completed the questionnaire. An overview of brucellosis, including causes, symptoms, potential ways of transmission, treatment, and infection prevention approaches for both humans and animals, was included. The structured questionnaire’s primary data were compiled and examined.

### Blood sample collection and serological test

The animals came from herds in the study areas and were not vaccinated. Breed, sex, age, source, and location were among the recorded animal parameters. In a 15 ml sterile tube, 10 ml of blood was aseptically taken from each animal’s jugular vein, allowed to clot, centrifuged for 15 min at 3,000 rpm, serum decanted, and kept at 20°C until testing. The Diagnostic Laboratory in Mandalay, Myanmar, handled all laboratory work. The Rose Bengal test (RBT) was used to analyze serum samples. The test was conducted using the RBT antigen, which is a standardized *B. abortus* antigen obtained from the Animal and Plant Health Agency (APHA), Surrey, UK. A stick applicator was used to thoroughly mix equal volumes (30 μl) of antigen and test sera, including the positive control sera, on a plate, which was then rocked for four minutes. Within one minute, agglutination received a score of ++, whereas beyond 1 min, it received a score of +. After four minutes, the absence of agglutination was classified as negative (−). An animal was considered positive if its brucellosis test result was positive (RBPT +). Similarly, herds were considered to have been brucellosis-positive if they contained one or more seropositive animals [16].

### Statistical analysis

Data were imported into STATA (version 17.0; Stata Corp., College Station, TX, USA) for analysis after being entered, organized, and coded using Microsoft Excel 2019. Descriptive statistics were employed as the primary research method to comprehend the key features of migratory farmers. This cross-sectional study allowed the identification of potential risk or protective factors in the presence of positive results for bovine brucellosis using the RBT test [16].

This study assumes that farm characteristics and migratory farm practices in the study area are associated with Brucella seropositivity on farms. Through the methodology proposed in this study, insight was obtained on the socioeconomic factors that impact access to veterinary information and brucellosis knowledge of farmers. The study’s null hypothesis stated that there was a lack of correlation between Brucella seropositivity at the farm level and the socioeconomic characteristics of farmers. Therefore, it is assumed that all farmers are aware of the disease regardless of their age, education, and income.

Several studies have used probit analysis to evaluate the adoption of agricultural technologies. Using this methodology, Jenkins evaluated factors affecting cotton farmers’ adoption of different information sources, including private media and agricultural extensions, and Gillespie examined factors affecting the adoption of four breeding technologies in the production of hogs [17,18]. Based on these findings, it is deemed appropriate to utilize a probit framework to increase estimation modeling efficiency. To assess the correlation between various variables and disease situations, univariate analysis was conducted. Age, education, income source, experience, herd size, migration factors, cattle abortion cases, and veterinary assistance were among the characteristics examined.

An empirical model can be described as follows:

*Y_i_* = β_1_+β_2_*x*_1_+β_2_*x*_2_+...+*u* (Formula)

where *i* = farm id; *Y_i_* = 1, if the farmer has the Brucella seropositivity at farm level (0 otherwise); *x_i_* = Vector representing factors affecting the farmers’ knowledge of the disease and access to veterinary; *β* = unknown parameters vectors; and *u* = error term.

## Results

### Farmer and farm characteristics

Of the 115 farmers, 95.65% were male, with a median age of 42 years (range, 23–75 years) and an average family size of four (range, 2–12 people). Most (81.74%) had completed only primary school, 16.52% had completed secondary school or higher, and 1.74% had no formal education. Most farmers (72.17%) raised cattle as their primary occupation, getting 86.69% of their total income, with an average of 8 years of experience. Other farmers (26.96%) were engaged in agricultural work and cattle farming, and 8.70% worked outside the farm. Almost half of the migratory farmers (49.57%) had monthly veterinary access, whereas 43.48% had received animal husbandry training (Table 1).

**Table 1.** Description of variables used in the analysis (Farmers’ Characteristics; *n =* 115).

Variables		Unit: Description	Mean
Farmer’s age		Years	42 (23–75)
Farmer’s Gender		1 = male; 0 = Female	95.65%
Farmer’s education	none	1 = none; 0 = other education	1.74%
	primary	1 = primary; 0 = other education	81.74%
	Secondary or higher	1 = secondary or higher; 0 = other education	16.52%
Number of family members		Person	4.71 (2–12)
Farmer’s Main job	Cattle farming	1 = cattle raising; 0 = other job	72.17%
	Cattle+Cropping	1 = cattle+cropping; 0 = cattle+other job	26.96%
	Cattle+Other job	1 = cattle+other job; 0 = cattle+agriculture	8.70%
Farmer’s income	Cattle farming	1 = income percentage from cattle farming; 0 = other income	86.69%
	Cropping	1 = income percentage from cropping; 0 = other income	8.21%
	Other job	1 = income percentage from other job; 0 = other income	4.83%
Cattle Raising Experience		Years	8 (6–10)
Vet Access	Quarterly	1 = farmer access veterinary service quarterly; 0 = no access	42.61%
	Monthly	1 = farmer access veterinary service monthly; 0 = no access	49.57%
	Weekly	1 = farmer access veterinary service weekly; 0 = no access	7.02%
Training	Husbandry	1 = farmer get animal husbandry training; 0 = no training	43.48%
	Disease control	1 = farmer get disease control training; 0 = no training	17.39%

### Seroprevalence of bovine brucellosis in cattle herd under migration practices

Five (4.34%) of the 115 serum samples resulted positive in RBT for Brucella antibodies. (Table 2). At the farm level, a higher prevalence (16.67%) was reported in small-scale farms (<10 heads), whereas large-scale farms (>20 heads) had the lowest prevalence (1.39%).

**Table 2.** Description of variables used in the analysis (Farm’ Characteristics).

Variables		Unit/Description	Mean
Total Cattle raised per farm		heads	32 (4–203)
Feeding system	Mixed/concentrate	1 = mixed feed/commercial concentrate; 0 = other feed	32.17%
	Roughage	1 = roughage: rice straws and agri-byproducts; 0 = other feed	6.09%
	Pasture	1 = pasture grass; 0 = other feed	61.74%
Cattle selling during migration	Live market	1 = sell cattle at live market; 0 = no sell	2.61%
	Other farms	1 = sell cattle to other neighbor farms; 0 = no sell	29.56%
Migration reason	Flood	1 = to avoid flood area; 0 = other	39.13%
	Drought	1 = scarcity of feed and water; 0 = other	43.48%
	Pasture	1 = to find pasture; 0 = other	64.35%
Migration distance		Kilometer	27.8 (15–76)
Migration duration		Month	6.21 (5.96–6.45)
Pay rent		1 = pay rent for migratory land; 0 = free charge	33.04%
Abortion cases		1 = number of abortion cases past 1 year; 0 = no abortion case	13.91%
Brucella seropositivity at farm level		1 = positive; 0 = negative	4.35%
	Small-scale	1 = positive in farms (<10 heads); 0 = negative	16.67%
	Medium-scale	1 = positive in farms (>10 heads); 0 = negative	4.00%
	Large-scale	1 = positive in farms (>20 heads); 0 = negative	1.39%

### Migration by purpose and season

**Figure 2.**
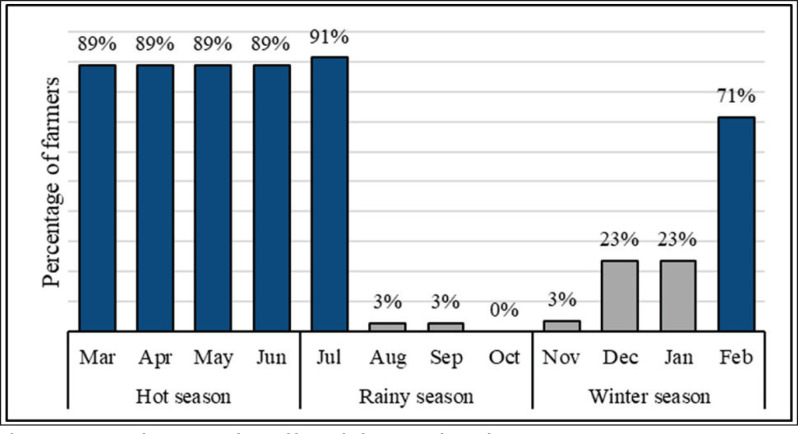
Migration by season and period by cattle farmers in the study area.

In the study of migratory farms, the households raise an average of 32 heads in a farm (range, 4–203 heads). The main purpose of the migration was to find greener pastures, which accounted for 64.35% of all farmers, followed by drought (43.48%) and flood in the native area (39.13%), as shown in Table 2. The percentage of cattle moved by season varied among farmers. During the hot season (March to June), there were more migrations (March to June): 89% in each month and winter season (November to February); in particular, 91% occurred in July. Farmers did not migrate their cattle to other places during October (Fig. 2).

**Figure 3.**
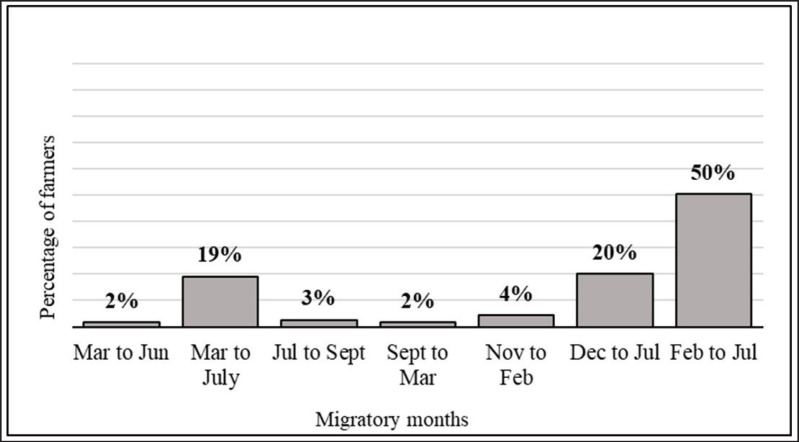
Migration periods by cattle farmers in the study area.

**Figure 4.**
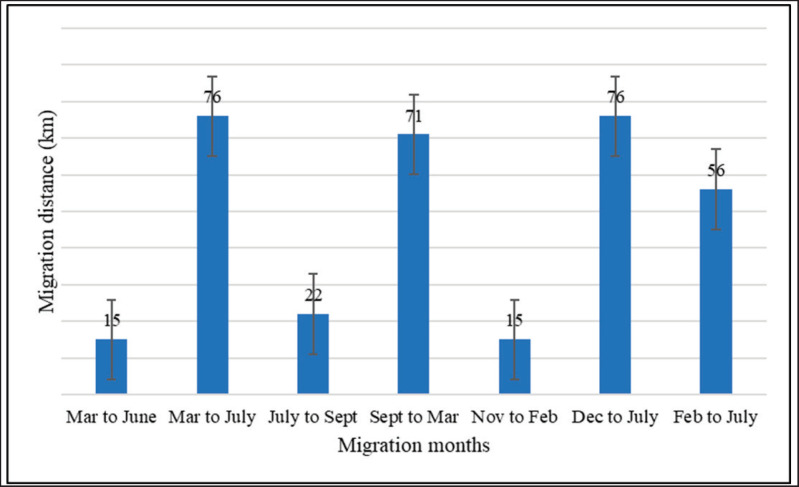
The length of cattle migration routes varies with the season; during various migration months, the distances range from 15 km to 76 km.

Note: Google Map distance measurements based on the cross-sectional survey were carried out in research areas in 2022 were used to determine the migration distance.

**Figure 5.**
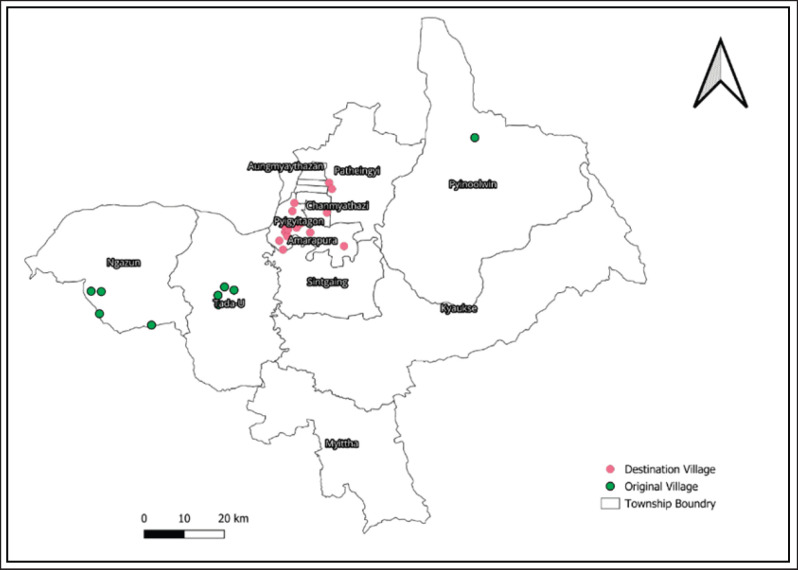
Map of the study area’s cattle farmers’ migration routes. Their permanent residences are shown by the green dots, whereas the locations of their seasonal movement are shown by the red dots.

Note: Farmers’ location information from a cross-sectional survey conducted in 2022 was used as an basis for QGIS Mapping.

During the winter season, 71% of the farmers stayed in the migration areas only in February, while 91% stayed in July. During the hot season (March to June), 89% of the migratory farmers used to relocate the cattle herds to other areas for pasture. As indicated in Figure 3, 50% of the farmers moved to the pasture area from February to July for 6 months, 20% followed from December to July (8 months), and 19% from March to July (5 months). The average duration of migration was 6.21 months (ranging from 5.96 to 6.45 months), with a migrated average of 27.8 km from their native places (15–76) (Table 2, Figs. 3–5).

### Potential brucellosis risk factors at the farm level

In the Probit regression model, the Brucella seropositivity in cattle farms was affected by several factors such as more family members (*p* < 0.01), having less farming experience (*p* < 0.01), raising fewer cattle (*p* < 0.01), a history of cow abortion (*p* < 0.01), longer migration distances (*p* < 0.01), selling cattle during migration (*p* < 0.01), and limited access to veterinary services (*p* < 0.01). The migratory decisions of farmers are influenced by their young age (*p* = 0.014) and higher level of education (*p* < 0.01). Based on these factors, our study indicated that small cattle farms that engage in migratory practices are more likely to get Brucella seropositivity (Table 3).

**Table 3.** Brucellosis prevalence and migratory farmers’ characteristics (*n =* 115).

Variable	Coef.	SE	p value
Farmer’s age	–0.125	0.051	0.014**
Farmer’s education	5.697	1.653	<0.01***
Family members	1.344	0.281	<0.01***
Percentage of cattle farming income	1.954	1.788	0.274
Cattle raising experience	–0.3412	0.067	<0.01***
Total cattle raised	-0.277	0.0666	<0.01***
Migration distance (km)	0.119	0.030	<0.01***
Abortion case past 1 year	9.398	1.765	<0.01***
Cattle selling during migration period	5.493	1.463	<0.01***
Monthly veterinary access	–4.047	0.631	<0.01***
Constant	–1.777	2.495	–0.71

** and *** denote statistical significance at the 5% and 1% level, respectively. Dependent variable, Brucella seropositivity: Yes (1); No (0).

## Discussion

In three regions of Myanmar’s dry zone, the seroprevalence of brucellosis in migratory farms as well as other risk factors at the farm level, were investigated. According to our findings, the prevalence of brucellosis was 4.34% at the farm level and 16.67% among smallholder cattle farmers. In comparison to neighboring countries of Indonesia and Thailand, where rates are 27.4% and 24.1%, respectively, Malaysia’s farm-level Brucella seropositivity among cattle is 21.8% (95% CI, 21.01–22.59) [19–21].

This study aimed to conduct the first analysis of cattle migration practices in Central Myanmar. Three areas in Myanmar were investigated for cattle migration during the COVID-19 pandemic and periods of political crisis. A descriptive analysis of cattle migration revealed its characteristics at the area level. The reasons for migration, as well as farmers’ individual characteristics and local climate, contribute to the migration patterns of farmers. These findings indicate a year-round migration, except in October, and a seasonally determined migration that occurred between March and July (Fig. 2) as a result of pasture shortages.

Local cropping patterns and seasons have an impact on grazing practices. Cattle in the CDZ are housed in small herds (*n =* 4) [14], in paddocks in the farm’s backyard at night, and sent to paddy fields or common areas for grazing during the day during the dry season. Farmers start cultivating at the start of the rainy season, while, to reduce crop damage, animals are moved out of the cropping area for four to five months. The animals were then relocated to different areas where they could have unrestricted access to feed. Animals in free-grazing environments freely interact, breed naturally, and share pasture ground and water sources. The rainy season and the calving season occur simultaneously in the area [22].

In the present study, in line with the traditional household decision-making model described by [23], husbands make the decisions regarding the herd and the benefit of the family. However, the decision to migrate differs depending on the age of the head of the household: Younger farmers are more likely to decide to relocate than older ones [24]. In our results, young farmers were more willing to take family members together along the migratory route by an average of three persons, and they mostly had eight years of farming experience. Farmers let household members, including children, take cattle to pasture areas.

The factors of farmers’ age, education level, family size, and farming experience were strongly related to Brucella seropositivity at the farm level. Higher education levels and older age have been shown to offer protection against brucellosis [25]. Our findings may be related to elder farmers’ prior farming experience and good farm hygiene procedures, even though they are difficult to explain. According to this study, farmers who have experienced brucellosis in their herds were more likely to have learned about the disease from other farms and veterinary extensions. The prevalence of brucellosis on farms was shown to be unaffected by either veterinary access or farming experience. This might be related to the nonexistent or very limited biosecurity and disease control training.

Receiving veterinary services was also shown to be an aid against Brucella prevalence in cattle, according to the probit analysis. In herds under a veterinarian’s supervision, bovine brucellosis is less common. Probably as a result of better monitoring and preventive health measures such as hygienic practices and appropriate disposal of aborted materials. While unhygienic practices contribute to the spread of infection, good hygiene protects against brucellosis [26,27]. It is often known that a low prevalence of diseases is a result of providing sufficient animal health services. By educating livestock farmers about hygienic practices and farming techniques and increasing public awareness of the disease, veterinary extension is thought to be crucial in reducing the spread of zoonosis.

Farmers rely heavily on cattle for their livelihoods. Informal interviews conducted revealed that cattle were considered assets and sold when money was needed for customs, school fees, sickness, or injuries. Furthermore, there is evidence that farmers only sell sick animals or in case of urgent need rather than profit, which leads to the animals being kept for longer periods and raising the risk of contracting brucellosis [28].

According to previous studies, herd size is a critical part of the spread of Brucella in susceptible and infected animals [29]. Larger herds have a greater number of positive animals [30]. The results showed that a small number of animals that moved longer distances were more likely to be seropositive for brucellosis. Farmers have a culture of migrating their cattle into groups of five to ten, and small herds must be combined with larger herds to reach pasture areas. Additionally, during grazing, larger herds are more likely to be nomadic and hence more likely to come into contact with infected herds [31]. Due to the difficulty of finding enough grazing and drinking spots in a given area, due to the large number of cattle that farmers keep, the travel distance is larger during the dry seasons (Figs. 4 and 5).

According to the current study, cattle with a history of abortions have a strong correlation with Brucella seropositivity. The primary route of disease transmission is aborted material, as healthy animals come into contact with contaminants and either directly or indirectly spread the disease through their food and water. Therefore, it is crucial to handle and dispose of aborted material properly to stop the transmission of disease to both humans and animals [32]. Abortion history has been proposed as a major risk factor for Brucella infection in Sri Lanka [33] and other countries [26,34]. The results of Sagamiko et al. [35] and Derdour et al. [36], who found an association between brucellosis and a history of abortion in cattle, are consistent with the findings. In contrast, Asmare et al. [37], Fereig et al. [38], and Adabi et al. [39] showed no significant correlation between brucellosis and a history of abortion in cattle. These disparities could be explained by variances in the research areas’ agroecology, management practices, and environmental circumstances, all of which could contribute to the spread of different abortion causes [40].

Our findings suggest that Brucella seropositivity in an area can be predicted by migration distance, abortion history, and migration practices. These factors might be taken as indicators for determining high-risk locations where surveillance should be increased. To enhance the development of prevention strategies, ongoing animal movement control efforts must be put into place. The study’s originality resides in its finding of a strong correlation between the socioeconomic characteristics of migratory farmers and the epidemiology of brucellosis, which ought to be taken into account in future disease management programs. As a result, subsequent veterinary epidemiological research should carefully take the “farmer factor” into

consideration. Developing nations with various social, cultural, and economic contexts related to farming practices can also use the presented findings.

Compared to the number of herds tested, a limited quantity of blood samples was taken due to travel restrictions caused by the COVID-19 pandemic and limitations on the number of animals given access by the farmers. There is a possibility that the group is not representative of the population. Nevertheless, the sample made it possible to estimate the disease prevalence in the study area.

## Conclusion

The results showed a significant prevalence of brucellosis among small-scale cattle farms in the Myanmar CDZ that use migratory practices, notwithstanding the aforementioned study limitations and restrictions. Migration decisions are significantly linked to household heads’ age and education level. Households led by younger males were more likely to experience migration. Migration practices, traveled distance, and abortion history in the herd may be associated with Brucella seropositivity in the area. These factors may be utilized as indicators of locations that require stronger monitoring. Migratory farmers must have access to veterinary services and awareness programs to help them understand the risks associated with livestock migration. To expedite the brucellosis control strategy in Myanmar, farm animal health extension services must consider farmers’ social factors and ongoing animal movement control programs.

## List of abbreviations

°C, degree celcius

APHA, Animal and Plant Health Agency

CDZ, Central Dry Zone

km, kilometers

min, minutes

RBT, Rose Bengal test

SMHs, Small scale migratory herds, TADs, Transboundry animal diseases

μl, microleter
